# Longitudinal follow-up of autoimmune neutropenia in adults—a single-cohort experience

**DOI:** 10.3389/fimmu.2026.1956807

**Published:** 2026-10-08

**Authors:** Stanley Niznik, Shirley Frizinsky, Soad Hag Yehia, Ronen Shavit, Shiri Weinstein, Mudi Misgav, Lilach Bonstein, Nancy Agmon-Levin

**Affiliations:** 1 Clinical Immunology, Allergy and Urticaria Institute, Sheba Medical Center, Ramat Gan, Israel; 2 The Gray Faculty of Medical and Health Science, Tel Aviv, Israel; 3 National Hemophilia Center, Sheba Medical Center, Ramat Gan, Israel; 4 Blood Bank and Platelet Immunology Laboratories, Haifa, Israel; 5 Ruth and Bruce Rappaport Faculty of Medicine, Technion, Haifa, Israel

**Keywords:** autoimmune, Evans syndrome, immune cytopenia, inborn error immunity, neutropenia

## Abstract

**Introduction:**

Adult autoimmune neutropenia (AIN) is a rare and probably underrecognized immune cytopenia for which population-based adult incidence estimates are not established. Unlike the benign, self-limiting course seen in children, the adult trajectory is poorly defined. We evaluated its presentation, course, and the factors associated with the requirement for treatment.

**Methods:**

Retrospective study of adults with neutropenia (ANC <1,500/µL) and anti-neutrophil antibodies on granulocyte immunofluorescence testing (GIFT) at a tertiary center, 2015–2025. GIFT positivity was considered supportive, not sufficient. Two patients later found to have a clonal disorder were described but excluded from outcome analyses.

**Results:**

A total of 41 patients were included (87.8% female; mean age 32.3 ± 14.8 years, followed 7.0 ± 7.1 years). A systemic immune disorder was present in 23 (56.1%): autoimmunity in 21 (51.2%), inborn errors of immunity in 6 (15.0%). Of 39 patients analyzed for outcome, 18 (46.2%) required neutropenia-directed therapy. These presented more often with fever (38.9% vs 4.8%, p=0.015) and thrombocytopenia (50.0% vs 14.3%, p=0.035), had lower platelet nadirs (p=0.024), and were admitted for neutropenic fever more often (3.6 vs 0.3 admissions, p=0.005). Malignancy (12.8%) and death (5.1%) occurred almost exclusively in this group, although neither difference was significant. Both nadirs were associated with the requirement for therapy (AUC 0.807).

**Conclusions:**

Adult AIN is heterogeneous and frequently marks broader immune dysregulation. A substantial subgroup carries a real burden of neutropenic fever and hospitalization, identifiable at presentation by the depth of both nadirs. AIN in an adult warrants a structured immunological evaluation and risk-adapted follow-up.

## Highlights

- This is one of the largest cohorts of adult autoimmune neutropenia reported to date, redefining it as a frequent marker of systemic immune dysregulation rather than a benign cytopenia.
- Treatment was required in about half of the cohort, and the depth of the platelet and neutrophil nadirs was associated with that requirement.

## Introduction

Autoimmune neutropenia is an immune-mediated cytopenia (1), which can be either primary or secondary (when it is associated with systemic immune diseases), and can affect both the number and the function of neutrophils (2). Clinical courses are typically chronic and cyclical with increased risk of invasive infection when neutrophil counts fall below 500/µL^3^ (3).

Autoimmune neutropenia was described both in children and in adults. In children, the course seems to be self-limited and is mainly associated with benign infections despite severe neutropenia (4). In adults, the clinical course is more varied and tends to be more severe, although data are limited (5). Autoimmune neutropenia can be a part of Evans syndrome, a triad with immune-mediated thrombocytopenia (ITP) and autoimmune hemolytic anemia (AIHA) (6). Evans syndrome is known to be associated with substantial morbidity and extra-hematological immune manifestation (7), lower overall survival (7), and a 120-fold higher risk for inborn error of immunity (IEI) (8), autoimmune lymphoproliferative disease (ALPS), common variable immunodeficiency (CVID), and autoinflammatory disease (9, 10). Another difference between child and adult AIN is the antigenic targets of autoantibodies against neutrophils. In children, the most common targets are of the human neutrophil antigen (HNA) system, particularly HNA-1a and HNA-1b (known as NA1 and NAB) (11, 12). It seems that in adults, the antibodies have a broader specificity including pan-FcRIIIB (recently described as anti HNA-3) (13).

Lastly, in the pediatric population, therapy of AIN is less needed, while in adults treatment of AIN in general and resistant autoimmune neutropenia has been described only in small case series, in which G-CSF was typically used, as well as successful treatment with prednisone and anecdotal success of intravenous immunoglobulin and anti-CD20 therapy as second line (14). Currently, reports of clinical course in adult AIN are scarce, with almost no longitudinal data (15). Hence, in the current study, our objective was to assess the course of AIN in adults, with a focus on clinical presentation, management, and long-term outcomes.

## Methods

### Study population

We assembled a retrospective cohort of adults evaluated at the Sheba Clinical Immunology, Angioedema and Allergy Institute and/or the Hematology Department, between January 2015 and January 2025 for neutropenia with evidence of autoantibodies toward neutrophils. Neutropenia was defined as an absolute neutrophil count (ANC) below 1,500/µL. Data regarding patient age, sex, concurrent immune mediated disease, pregnancy status, and laboratory parameters and data regarding therapy, hospitalization, and hematological malignancies were retrieved from patient clinical files.

A total of 41 patients met the inclusion criteria and are described below. During follow-up, two patients—both older than 60 years at presentation—were diagnosed with clonal hematological disorders: T-cell large granular lymphocytic leukemia and myelodysplastic syndrome. Because anti-neutrophil antibodies were detected close to the diagnosis of clonal disease, a secondary cause for their neutropenia could not be confidently excluded. However, because their antibodies were identified using the same assay as the rest of the cohort, we did not consider the autoimmune mechanism irrelevant. Both patients were therefore retained in the initial cohort description and in **Table 1** but excluded from the treatment and outcome analyses, which are restricted to the remaining 39 patients (**Tables 2**–**4**, **Figure 1**).

**Table 1:** Autoimmune neutropenia general parameters (N = 41).

Parameter	Result	n assessed
Female rate	36 (87.8%)	41
Average age at diagnosis, years	32.3 ± 14.8	41
Follow-up duration, years	7.0 ± 7.1	41
Associated autoimmunity	21 (51.2%)	41
Associated inborn error of immunity (IEI)	6 (15.0%)	40
Any systemic immune disorder	23 (56.1%)	41
Diagnosis during pregnancy (of women)	6 (16.7%)	36
Fever at presentation	8 (19.5%)	41
Infection at presentation	2 (4.9%)	41
ANC nadir, cells/µL	363 ± 325	39
Age at ANC nadir, years	36.7 ± 13.0	38
Autoimmune hemolytic anemia (AIHA)	7 (17.5%)	40
Thrombocytopenia (<130,000/µL)	14 (34.1%)	41
Platelet nadir, ×10³/µL	154.8 ± 66.7	41
ANA positivity	15 (37.5%)	40
C3 nadir, mg/dL	100.8 ± 26.6	36
C4 nadir, mg/dL	26.3 ± 11.3	36

Percentages are calculated on the number of patients assessed for each variable. One patient whose C3 and C4 were recorded as zero was treated as missing.

**Table 2A:** Prognostic parameters (n=39).

Parameter	n=39
Hospitalization due to neutropenic fever	11 (28.2%)
Average number of hospitalizations	1.8 ± 4.0
Average number in hospitalized patients (n=11)	6.4 ± 5.5
Hematological malignancy (all)	5 (12.8%)
Lymphoid malignancy	4 (10.3%)
Myeloid malignancy	1 (2.6%)
Death	2 (5.1%)
No therapy required	21 (53.8%)
Granulocyte colony-stimulating factor	15 (38.5%)
Steroid therapy	12 (30.8%)
Both G-CSF and steroids	9 (23.1%)
Intravenous immunoglobulin	7 (17.9%)
B cell depletion therapy	5 (12.8%)
Mycophenolate mofetil	4 (10.3%)
Azathioprine	3 (7.7%)
Methotrexate	2 (5.1%)
Other targeted agents	11 (28.2%)
Splenectomy	1 (2.6%)

Immunoglobulin, B-cell depletion, and the other targeted agents were prescribed for an associated systemic disease or, in the immunodeficient patients, as replacement therapy; see Methods.

**Table 2b:** Hematological malignancy, per patient.

Diagnosis	Age at AIN, y	Age at malignancy, y	Interval	Group
Lymphoma in ALPS	19	20	1 year	Therapy
Lymphoma in ALPS-like disease	28	31	3 years	Therapy
Acute myeloid leukemia M6	39	51	12 years	Therapy
Mantle-zone lymphoma	32	45	13 years	No therapy
B-cell non-Hodgkin lymphoma	14	36	22 years	Therapy

The two patients excluded from the outcome analysis carried diagnoses of T-cell large granular lymphocytic leukemia and myelodysplastic syndrome; in neither case could the date of diagnosis be established, which is the reason for their exclusion (see Methods).

**Table 3:** Comparison of therapy group vs. non therapy group.

Parameter	Therapy group (n=18)	No therapy group (n=21)	P value
Age at presentation, years	31.6 ± 14.7	29.9 ± 12.2	0.699
Female sex	17 (94.4%)	18 (85.7%)	0.609
Associated autoimmune disease	9 (50.0%)	11 (52.4%)	1.000
Inborn error of immunity	3 (17.6%)	3 (14.3%)	1.000
Diagnosis during pregnancy	4 (26.7%)	2 (11.1%)	0.375
Fever at presentation	7 (38.9%)	1 (4.8%)	0.015
ANC nadir, cells/µL	245 ± 242	451 ± 370	0.059
Age at ANC nadir, years	37.7 ± 13.6	33.6 ± 10.3	0.328
Autoimmune hemolytic anemia	5 (27.8%)	1 (5.0%)	0.083
Thrombocytopenia (<130,000/µL)	9 (50.0%)	3 (14.3%)	0.035
Platelet nadir, ×10³/µL	134.2 ± 67.6	178.7 ± 59.8	0.024
ANA positivity	8 (47.1%)	6 (28.6%)	0.318
Hospitalizations, no. per patient	3.6 ± 5.4	0.3 ± 0.9	0.005
Hematological malignancy	4 (22.2%)	1 (4.8%)	0.162
Death	2 (11.1%)	0 (0%)	0.206

Denominators vary where data were missing for individual variables; percentages are calculated on the patients assessed.

**Table 4:** Predictive model of therapy requirement.

Predictor	Odds ratio (95% CI)	Coefficient	P value
Platelet nadir, per 10 ×10³/µL lower	1.145 (1.011–1.296)	0.135	0.032
Neutrophil nadir, per 100 cells/µL lower	1.269 (0.969–1.663)	0.239	0.083

Logistic regression, n=37 (two patients had no recorded ANC nadir). Odds ratios are expressed per unit decrease, so that a value above 1 indicates that a lower count is associated with a greater likelihood of therapy. McFadden pseudo-R² 0.188; likelihood-ratio p = 0.009. Area under the ROC curve 0.807 (95% CI 0.634–0.946), 0.785 after correction for optimism; sensitivity 87.5%, specificity 71.4%, positive predictive value 70.0%, negative predictive value 88.2%.

**Figure 1:**
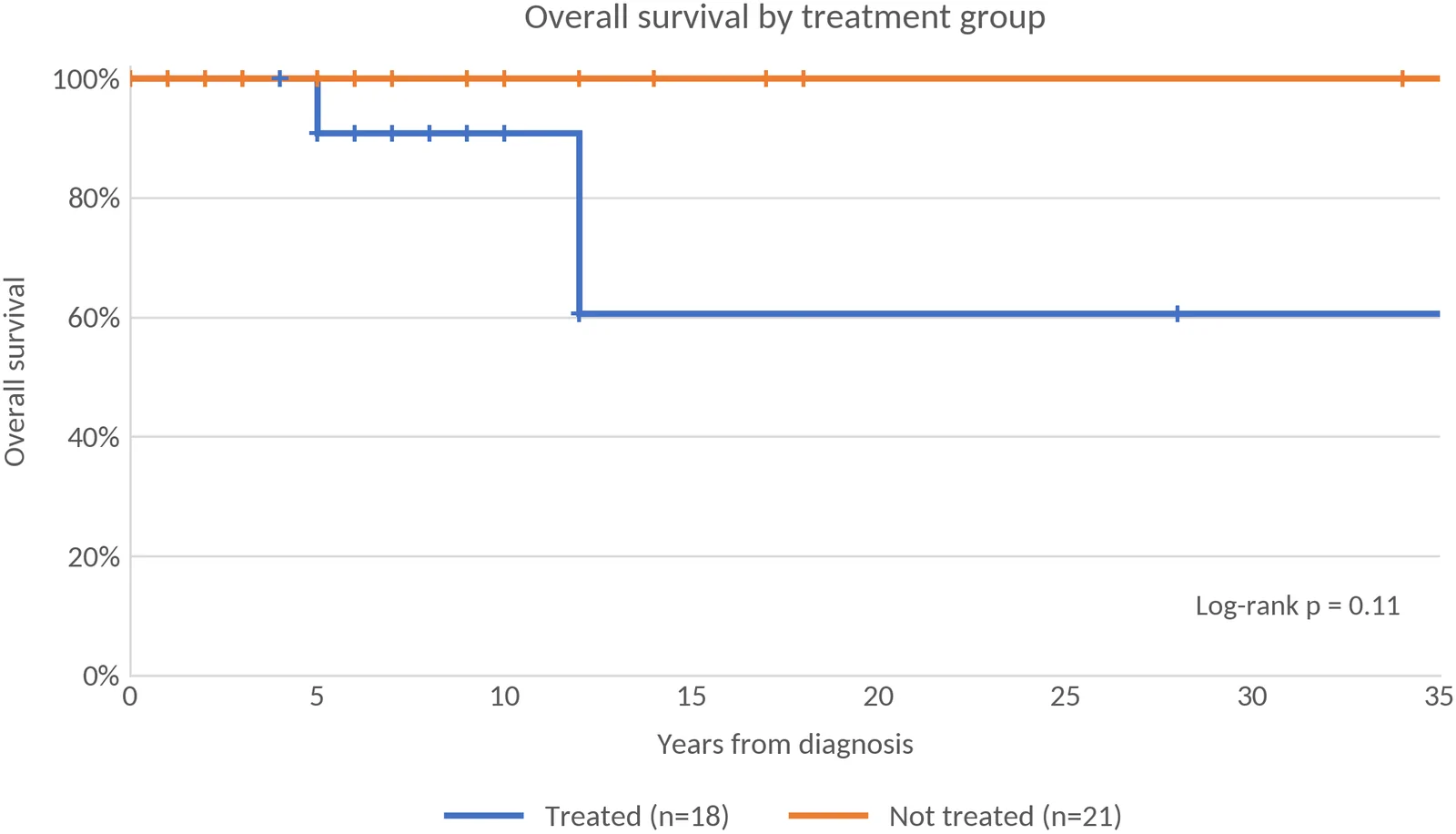
Overall survival according to treatment group (n=39). Numbers at risk at 0, 5, 10, 15, 20, 25, and 30 years: treated 18, 11, 4, 1, 1, 1, and 0; untreated 21, 11, 6, 3, 1, 1, and 1.

### Detection of anti-neutrophil antibodies

To evaluate for the presence of circulating anti-neutrophil antibodies, patient serum was analyzed using an indirect granulocyte immunofluorescence test (i-GIFT) coupled with flow cytometry. To ensure comprehensive coverage across variable human neutrophil antigens (HNAs), the serum was incubated with a standardized screening panel of freshly isolated granulocytes obtained from four healthy, phenotyped donors. Antibody binding to the donor cells was subsequently labeled using a fluorescein-conjugated secondary anti-human globulin, and the reaction was quantitatively assessed by fluorescence intensity using a flow cytometer; a significant rightward shift in fluorescence relative to negative controls designated a positive test result (16). A positive GIFT was considered supportive of autoimmune neutropenia rather than sufficient in isolation to establish etiology, and clinicians assessed alternative or contributing causes, including medication-related, infectious, nutritional, systemic autoimmune, LGL-associated, clonal or hematological, and genetic causes. Testing for HLA class I antibodies, which are also expressed on granulocytes and may react in the i-GIFT, was not performed routinely and was introduced only in more recent years; HLA antibody data (by lymphocyte immunofluorescence and/or panel-reactive antibody [PRA] testing) were therefore available for approximately half of the cohort. A contribution of anti-HLA reactivity to individual positive results could not be formally excluded (see Limitations).

### Treatment

Treatments were prescribed according to the treating physician assessment and not according to a study protocol. To group comparison, AIN-directed therapy was defined as treatment given specifically for the neutropenia, namely, corticosteroids and/or granulocyte colony-stimulating factor (G-CSF). Immunoglobulin was prescribed at replacement dose (0.5 g/kg monthly) in patients with an inborn error of immunity and at immunomodulatory dose (2 g/kg) in the treated group, and only the latter was regarded as treatment for AIN. Rituximab was given for hematological malignancy or for immune thrombocytopenia (two patients) and never for the neutropenia; in these patients, the ANC did not fall below 500/µL at any point. Other agents recorded in the cohort (belimumab, sirolimus, abatacept, secukinumab, tacrolimus, hydroxychloroquine, colchicine, and anakinra) were prescribed for an associated systemic disease. Patients receiving only these treatments were therefore classified as not having received AIN-directed therapy. Because laboratory severity may itself have influenced the decision to treat, the requirement for therapy was analyzed as a clinical-management outcome rather than as an independent definition of biological disease severity.

### Hematological malignancy

For every patient in whom a hematological malignancy was diagnosed, the precise diagnosis was recorded together with the age at which it was made, so that its temporal relationship to the neutropenia could be established (**Table 2b**).

### Statistical analysis

Continuous variables are presented as mean ± standard deviation and were compared using Welch’s t-test where the distribution allowed and the Mann–Whitney U test where it did not, with normality assessed by the Shapiro–Wilk test. Categorical variables are presented as n (%) and were compared using Fisher’s exact test throughout, given the small expected cell counts. Missing values were handled by available-case analysis, and the number of patients assessed for each variable is given in the tables. Factors associated with the requirement for therapy were examined by logistic regression; several models were compared, and the best-fitting model is reported. Model discrimination was assessed by the area under the ROC curve, with correction for optimism by bootstrapping, and sensitivity and specificity are reported at the optimal threshold. Overall survival was estimated by the Kaplan–Meier method and compared by the log-rank test. A two-sided p value below 0.05 was considered significant.

Study was done according to local ethical committee approval.

## Results

A total of 41 patients were included in our cohort (**Table 1**), with predominance of female (87.8%, 36/41), and a mean age at diagnosis of 32.3 years (± 14.8). Clinical follow-up averaged 7.0 years (± 7.1). Concurrent autoimmune disease was present in 21/41 (51.2%) of patients, including Hashimoto thyroiditis, SLE, APS, vitiligo, IBD, uveitis and Behçet disease. Additionally, 6/40 (15.0%) were eventually diagnosed with an inborn error of immunity (IEI), with two patients with common variable immunodeficiency (CVID), one of whom carries a heterozygous NFKB1 variant, one patient with autoimmune lymphoproliferative like disease (ALPS-like), one patient with dedicator of cytokinesis 2 (DOCK2) mutation, one patient with IgM deficiency, and lastly one patient with heterozygotic deficiency of Adenosine Deaminase 2. Four patients carried both an autoimmune disease and an IEI, so that some form of systemic immune disorder was documented in 23/41 (56.1%) of the cohort.

Clinical presentation (**Table 1**) varied with 6/36 (16.7%) of the female subgroup diagnosed during pregnancy, while 8/41 (19.5%) presented with fever, and 2/41 (4.9%) presented with infection. In this cohort, many were diagnosed with AIN as an incidental finding; nonetheless, the average neutrophil nadir count was 363 ± 325/µL. Regarding other laboratory findings, 15/40 (37.5%) were ANA positive, 14/41 (34.1%) had thrombocytopenia at any point during follow-up, and 7/40 (17.5%) were diagnosed with autoimmune hemolytic anemia (AIHA). The mean platelet nadir was 154.8 ± 66.7 ×10³/µL, and complement nadirs were within the normal range (C3 100.8 ± 26.6 mg/dL and C4 26.3 ± 11.3 mg/dL).

As detailed in the Methods, two patients were subsequently found to have a clonal hematological disorder whose temporal relationship to the neutropenia could not be established. Both are included in the description above, and in **Table 1**, but were not carried into the analysis of treatment and outcomes. The results that follow therefore refer to 39 patients.

### Prognostic parameters and outcomes

Four prognostic outcomes were evaluated (**Table 2A**): requirement of therapy, hospitalization for neutropenic fever, hematological malignancy, and mortality.

- Therapy: 18/39 (46.2%) required systemic intervention. G-CSF was used in 15/39 (38.5%) and prednisone in 12/39 (30.8%), while 9/39 (23.1%) required both. Among the G-CSF recipients, a hematological response was documented in 6 of the 11 patients in whom response was recorded, although response was not defined prospectively and is reported descriptively.
- Mortality: The overall mortality rate was 5.1% (2/39). Both deaths occurred among patients who required therapy (11.1% vs 0% in controls, p = 0.206), and both were due to infection, one following surgery and one spontaneous. Both patients carried a hematological malignancy at the time of death.
- Hospitalization: A total of 11 patients (28.2%) were admitted at least once with neutropenic fever. The average number of hospitalizations was significantly higher in the therapy group (3.6 ± 5.4 vs 0.3 ± 0.9 admissions per patient, p = 0.005), and among those ever hospitalized, the mean was 6.4 ± 5.5.
- Malignancy: Hematological malignancies occurred in 5/39 (12.8%) of patients, including two B-cell non-Hodgkin lymphomas, one acute myeloid leukemia (M6), and lymphoma arising in the two patients with ALPS and ALPS-like disease. The interval between the diagnosis of neutropenia and that of the malignancy ranged from 1 to 22 years (**Table 2b**). Malignancy occurred more frequently in the therapy group (22.2% vs 4.8%), although with these numbers, the difference did not reach statistical significance (p = 0.162).

When comparing patients who required therapy to those managed conservatively (**Table 3**), the groups were similar in age, sex, and ANA positivity, as well as in associated autoimmunity and inborn errors of immunity. The therapy group, however, demonstrated a more severe clinical phenotype, defined by fever at presentation (38.9% vs 4.8%, p = 0.015), thrombocytopenia (50.0% vs 14.3%, p = 0.035), and a lower platelet nadir (134.2 ± 67.6 vs 178.7 ± 59.8 ×10³/µL, p = 0.024). The neutrophil nadir was lower in the same direction but fell just short of significance (245 ± 242 vs 451 ± 370/µL, p = 0.059), as did the presence of AIHA (27.8% vs 5.0%, p = 0.083).

To identify parameters associated most with the requirement for therapy, we ran a multivariate analysis (**Table 4**). Several models were examined, and the combination of platelet nadir and neutrophil nadir fitted the data best. Every decrease of 10 ×10³/µL in the platelet nadir was associated with a 15% increase in the likelihood of therapy (OR 1.145, 95% CI 1.011–1.296, p = 0.032), and every decrease of 100 cells/µL in the neutrophil nadir with a 27% increase (OR 1.269, 95% CI 0.969–1.663, p = 0.083).

This dual-parameter profile demonstrated reasonable discriminative accuracy (AUC 0.807, 95% CI 0.634–0.946, falling to 0.785 after correction for optimism), with a sensitivity of 87.5% and a specificity of 71.4% at the optimal threshold, outperforming either marker alone. Given the size of the cohort, the absence of external validation, and the fact that the outcome reflects a clinical decision rather than a biologically defined phenotype, we regard this model as exploratory.

Overall survival according to treatment group is shown in **Figure 1**. No deaths occurred among the patients managed conservatively, and the curves separate after the fifth year of follow-up; with only two events, however, the difference did not reach statistical significance (log-rank p = 0.11).

## Discussion

This study brings to light adult autoimmune neutropenia (AIN), a frequently overlooked entity within the landscape of immune cytopenias. By utilizing longitudinal data from 41 adult patients with a robust average follow-up of approximately 7 years, we were able to reevaluate the clinical parameters and long-term outcomes of this unique population.

The inception of this study grew from clinical need. As clinicians, we have encountered two types of patient: one with the familiar, benign form of autoimmune neutropenia, in which the neutropenia carries no clinical relevance, and another altogether different, requiring repeated immunomodulation and repeated admission, and running a considerably less benign course. Current guidance, however, speaks mainly to the first of these. The European consensus on chronic neutropenia recommends that the decision on continuous G-CSF rest on the history and severity of infection rather than on the neutrophil count itself, and advises against antibiotic prophylaxis in any type of neutropenia (17, 18). Immunomodulation aimed at the neutropenia is not part of that arsenal: corticosteroids and comparable agents are not generally recommended, and where immunosuppression is advised, it is directed at an associated systemic disease rather than at the neutrophil count (17, 18). In practice, this leaves a gap. Out of our 39 patients, 12 (30.8%) received corticosteroids and 9 (23.1%) received both corticosteroids and G-CSF, yet the published experience of immunomodulation in adult AIN is confined to small case series that state neither dose, nor duration, nor which patient is likely to need it. It was this gap that our cohort was assembled to describe.

This brought to light several notions, which to the best of our knowledge are not common even in the immunohematology field. First, patients are young (32.3± 14.8 years) and with female predominance; this is similar in nature to ITP (19) and AIHA (20), but with a larger extent—87.8% of our cohort was female; we believe this relates to bias of selection given our work with young females and a small cohort, and we have no reason to believe AIN has a different epidemiology than the other immune cytopenias Second, AIN is not an isolated disease; in 56.1% of our cohort, AIN was linked with systemic immune-mediated conditions, namely, 51.2% of patients were diagnosed with autoimmune diseases, and a surprising 15.0% were linked to inborn errors of immunity (IEI). The latter finding has not been documented in previous cohorts and may be on the one hand the result of a referral bias inherent to tertiary immunology institute while on the other the extended meticulous immune assessment which evolved in the recent decade and enabled better detection of IEI.

To address our objective, patients were stratified according to the clinical decision to initiate treatment. While inherently subject to provider discretion, this pragmatic approach reflected real-world clinical judgment in managing this cohort of predominantly young female patients. Notably, treated patients presented significantly more often with neutropenic fever—a complication infrequently highlighted in autoimmune neutropenia (AIN) and more typically described in oncology populations.

Although severe neutropenia in AIN is widely regarded as running a relatively benign clinical course, our findings demonstrate that a clinically meaningful subset of patients fails to adequately mobilize neutrophils during infectious episodes. Consequently, this impaired reserve was associated with a markedly higher rate of hospital admissions (mean 3.6 vs. 0.3 hospitalizations, *p* = 0.005). Given the young age of this cohort, this finding not only highlights a substantial burden on quality of life and healthcare utilization but also supports the potential pathogenic relevance of anti-neutrophil antibodies in mediating infectious susceptibility.

Conversely, hard endpoints such as subsequent malignancy and overall mortality were only numerically higher in the treated cohort, without reaching statistical significance. Larger, multicenter prospective studies are warranted to definitively determine whether this clinically aggressive subset of AIN confers an increased long-term risk of malignancy or excess mortality.

We further tried to evaluate what would predict these patients; in multivariate analysis, the combination of platelet nadir and ANC nadir fitted the data best with an AUC of 0.807 with a sensitivity of 87.5% and a specificity of 71.4% at the optimal threshold, outperforming either marker alone. Interestingly, in other autoimmune conditions, bicytopenia may also be a sign of a more severe course (21, 22); whether bicytopenia in the context of AIN is a sign of a more systemic disease or a more severe disease, only a future properly designed cohort will be able to tell. Taking this altogether, it seems that adult AIN may be a non-benign harmful condition that requires prudent clinical workup, assessing for systemic immune manifestations and tailored therapy.

Our study is not without limitations, most notably its retrospective design and the potential for selection bias within a tertiary referral center. Furthermore, our local healthcare structures do not cover AIN evaluation (e.g., GIFT study), which may bias the cohort toward patients with either higher financial means or symptoms severe enough to justify out-of-pocket expenditure. Finally, for some patients, especially those with no other cytopenia, it is difficult to definitively determine whether the observed prognosis is driven by the immune-mediated mechanism or the neutropenia itself. Given the scarcity of these patients, our cohort reflects real-world conditions and strengthens the need for a multicentric, international effort of adult patients with AIN. A further limitation concerns anti-HLA class I antibodies, which bind granulocytes and may produce reactivity in the i-GIFT; this is particularly relevant in a predominantly female cohort that included pregnancy-associated diagnoses, in which alloimmunization is common. HLA antibody testing was not performed routinely, was introduced only in more recent years, and was available for approximately half of the cohort; given the cost of the assay, it was not extended to the remaining patients, and a contribution of anti-HLA reactivity to individual GIFT results therefore cannot be excluded.

## Conclusions

In this longitudinal cohort, adult autoimmune neutropenia (AIN) emerged as a heterogeneous entity that frequently diverges from its classical depiction as an exclusively benign cytopenia. While a subset of patients follows an indolent course without requiring intervention, our findings delineate a clinically vulnerable subgroup characterized by impaired neutrophil reserve during infection, recurrent neutropenic fever, and a substantial burden of hospitalizations. The co-occurrence of severe neutrophil and platelet nadirs serves to help identify the predictive framework for identifying patients prone to a more aggressive clinical trajectory. Furthermore, the frequent association of AIN with systemic autoimmunity and inborn errors of immunity underscores that adult AIN often acts as a sentinel manifestation of broader immune dysregulation rather than an isolated hematological anomaly. Comprehensive baseline immunological evaluation and risk-stratified management are essential to optimize outcomes in this population.

## Data Availability

The raw data supporting the conclusions of this article will be made available by the authors, without undue reservation.
